# Genetic suppressors of *Δgrx3 Δgrx4*, lacking redundant multidomain monothiol yeast glutaredoxins, rescue growth and iron homeostasis

**DOI:** 10.1042/BSR20212665

**Published:** 2022-06-09

**Authors:** Guichun Li, Ankanahalli N. Nanjaraj Urs, Andrew Dancis, Yan Zhang

**Affiliations:** 1 Tianjin Key Laboratory for Modern Drug Delivery & High-Efficiency, Collaborative Innovation Center of Chemical Science and Engineering, School of Pharmaceutical Science and Technology, Tianjin University, Tianjin 300072, China; 2 Frontiers Science Center for Synthetic Biology (Ministry of Education), Tianjin University, Tianjin 300072, China; 3 Department of Pharmacology, Physiology, and Neuroscience, New Jersey Medical School, Rutgers University, Newark, New Jersey, U.S.A.; Division of Hematology-Oncology, Department of Medicine, Perelman School of Medicine, University of Pennsylvania, Philadelphia, Pennsylvania, U.S.A.

**Keywords:** Aconitase, Ferric reductase, Iron metabolism, Iron-sulfur, Monothiol glutaredoxins, Suppressor mutation

## Abstract

*Saccharomyces cerevisiae* Grx3 and Grx4 are multidomain monothiol glutaredoxins that are redundant with each other. They can be efficiently complemented by heterologous expression of their mammalian ortholog, PICOT, which has been linked to tumor development and embryogenesis. PICOT is now believed to act as a chaperone distributing Fe-S clusters, although the first link to iron metabolism was observed with its yeast counterparts. Like PICOT, yeast Grx3 and Grx4 reside in the cytosol and nucleus where they form unusual Fe-S clusters coordinated by two glutaredoxins with CGFS motifs and two molecules of glutathione. Depletion or deletion of Grx3/Grx4 leads to functional impairment of virtually all cellular iron-dependent processes and loss of cell viability, thus making these genes the most upstream components of the iron utilization system. Nevertheless, the *Δgrx3/4* double mutant in the BY4741 genetic background is viable and exhibits slow but stable growth under hypoxic conditions. Upon exposure to air, growth of the double deletion strain ceases, and suppressor mutants appear. Adopting a high copy-number library screen approach, we discovered novel genetic interactions: overexpression of ESL1, ESL2, SOK1, SFP1 or BDF2 partially rescues growth and iron utilization defects of *Δgrx3/4*. This genetic escape from the requirement for Grx3/Grx4 has not been previously described. Our study shows that even a far-upstream component of the iron regulatory machinery (Grx3/4) can be bypassed, and cellular networks involving RIM101 pH sensing, cAMP signaling, mTOR nutritional signaling, or bromodomain acetylation, may confer the bypassing activities.

## Introduction

Organisms have evolved sophisticated mechanisms to maintain iron homeostasis [1,2]. Extensive work in the budding yeast *Saccharomyces cerevisiae* has uncovered the iron regulatory networks that control iron metabolism. The transcription factor Aft1/2 plays a key role in sensing cellular iron status and communicating this to the iron regulon, a set of iron responsive genes involved in the uptake, compartmentalization, storage, intracellular trafficking and utilization of iron. The coupling to the iron signal occurs via a complicated and clever mechanism, only recently defined [3]. Briefly, a regulatory Fe-S cluster is formed on the CDC residues of Aft1/2 in response to a cellular signal of iron availability. This sets in motion a sequence of events according to which the transcription factor is removed from its DNA bindings sites in the nucleus, dimerized, and exported to the cytoplasm. At this point, the Fe/S cluster containing Aft1/2 factor is thought to be silent for its regulatory roles, for only the apo form binds to cognate DNA sequences of the iron regulon, turning on cellular iron uptake and turning off high utilization components. The regulatory Aft1/2 Fe/S cluster originates on Grx3/4 glutaredoxins, and it is then transferred to Aft1/2-BolA complexes, and finally transferred to the Aft1/2 CDC motif [4–6]. Aft1/2 forms a physical protein–protein interaction with Grx3/4 during the Fe/S cluster transfer step [7].

Yeast Grx3 and Grx4 are members of the multidomain monothiol glutaredoxin (Grx) family, conserved in organisms ranging from bacteria to humans. These proteins display an N-terminal Trx (thioredoxin-like) domain and a C-terminal Grx (glutaredoxin- like) domain [8]. The putative active site in the Grx domain contains a highly conserved CGFS motif, specifically required for liganding an Fe-S cluster that is critical for controlling Aft1/2 subcellular localization. Grx3 and Grx4 perform redundant functions with each other; deletion of each single gene has little effect on iron regulation, while deletion/depletion of both Grx3 and Grx4 leads to constitutive expression of iron regulon genes [9]. In addition to the role in regulating iron homeostasis through Aft1/2, it has been proposed that the Fe-S cluster formed by the dimeric Grx3/4 constitutes the labile iron pool that can be mobilized for use in iron-dependent enzymes in various cellular compartments, including mitochondria, cytoplasm and nucleus. For example, aconitase, which reversibly catalyzes the conversion of citrate to isocitrate in the tricarboxylic acid cycle in mitochondria and requires a [4Fe-4S] cluster for its activity, depends on intact Grx3/4 [10]. Grx3/4-deficient cells develop severe defects in both mitochondrial and cytosolic iron-dependent processes despite the induction of the Aft1-dependent iron uptake system [7,11].

Fe-S cluster biogenesis takes place in carefully choreographed stages, with the initial step taking place in mitochondria and involving a cysteine desulfurase [12], and formation of a 2Fe-2S cluster intermediate on a protein scaffold [13–15]. Subsequently an intermediate or intermediates is exported to the cytoplasm via Atm1, an ATP-dependent transporter in the mitochondrial inner membrane [16,17]. The intermediate, which has never been purified or chemically characterized, is required for cytoplasmic Fe-S cluster assembly and tRNA thiolation [18]. Various cytoplasmic/nuclear biosynthesis factors termed cytosolic sulfur cluster assembly (CIA) mediate synthesis of the Fe-S clusters in these compartments [19,20]. Although biosynthesis of Grx3/4 clusters requires mitochondrial Fe-S cluster assembly to generate the Atm1 exportable intermediate, CIA components are not involved. Thus the biogenesis of Grx3/4 Fe/S clusters can be placed far upstream in the overall scheme but after the mitochondrial and mitochondrial export activities.

In some *S. cerevisiae* genetic backgrounds (e.g., W303), deletion of Grx3 and Grx4 is lethal, while in other backgrounds (e.g., BY4741/2), the double deletion mutant is viable albeit slow growing. In all backgrounds, iron uptake pathways are activated and iron utilization pathways are severely impaired in the *Δgrx3/4* mutants, as expected from negative effects on the Aft1/2 CDC cluster [21]. We also observed that cultures of the *Δgrx3/4* strain exhibit genetic instability, similar to *∆yfh1* mutants [22–24], accumulating suppressors with high frequency. Thus there must exist one or more bypass mechanisms for rescuing growth of cells lacking Grx3 and Grx4. Motivated by these initial findings, a genetic screen was performed to identify *Δgrx3/4* suppressors using a high copy-number plasmid library. Overexpression of ESL1, ESL2, SOK1, SFP1 or BDF2 was found to restore the growth of *Δgrx3/4*, and was associated with partial rescue of iron homeostasis in the mutant. A more detailed analysis of how these aforementioned genes confer bypass activity is presented in the Discussion.

## Materials and methods

### Growth media

The standard non-selective rich growth media was composed of 1% yeast extract, 2% peptone, 100 µg/ml adenine. In most experiments, 2% glucose was used as the carbon source (YPAD). Selective defined media was composed of 6.7 g/L yeast nitrogen base without amino acids from Difco, 2% glucose, CSM, or CSM with amino acid dropout from MP Biomedical, and 1.5% Difco agar as a solidifying agent for agar plates.

### Yeast strains and crosses

Yeast strains used in the present study are listed in Supplementary Table S1. If not stated otherwise BY4741 and BY4742 are the parental strains. Sporulation and tetrad dissection of *Δgrx3/4* diploids were performed according to published methods [25,26]. Briefly, GRX3/*Δgrx3* GRX4/*Δgrx4* diploid strains were grown on YPAD agar at 30°C for 2–3 days. A swab of cells was patched onto pre-spore media (0.8% yeast extract, 0.3% peptone, 2% agar and 40% glucose) and grown at 30°C for 2 days. Further, cells from pre-spore plates were patched on to sporulation media composed of 0.25% yeast extract, 1.5% potassium acetate, 2% agar, 0.05% glucose, and 1× spore amino acids (Supplementary Table S2) and grown at 30°C. Diploid cells sporulated in 4–5 days and were then subjected to tetrad analysis. For tetrad dissection, cells were scraped from sporulation plate using a sterile stick, and gently suspended in 40 µl of zymolyase (1 mg/ml in 1 M sorbitol) and incubated for 15 min at 30°C. The cells were diluted gently by adding 500 µl of sterile deionized water. Using a sterile wire loop, the cell suspension was mixed gently and a loopful of cells was spread in a line on a YPAD plate. The tetrads were dissected using Nikon eclipse Ci Microscope. Crosses of haploid strains were performed by micromanipulation of zygotes [25].

### Plasmids and primers

All plasmids and primers used in the present study are listed in Supplementary Tables S3 and S4. Plasmid clones used in the present study were constructed by Gibson Assembly [27] and were confirmed by DNA sequencing.

### Genetic screen for high copy number *Δgrx3/4* suppressors

The genetic screen was performed using a YEp13-based yeast high copy-number genomic library (ATCC 37323). A haploid yeast shuffle strain was generated (strain no. 124-67), carrying *Δgrx3 Δgrx4* deletions, a ura3-52 marker, and a plasmid with functional GRX3 and URA3 (pRS416-GRX3-HA) allowing counter-selection with 5-fluoroorotic acid (FOA) medium. The shuffle strain was transformed with a genomic library using lithium acetate transformation, and transformants were selected for leucine prototrophy [28]. The transformants were replica plated to FOA medium to select against the URA3-containing pRS416-Grx3 plasmid. Most transformants were non-viable following the FOA treatment, but a few grew and some grew robustly. These were retained and plasmids from the viable yeast colonies following counter-selection were isolated and transformed into *Escherichia coli* DH5α strain to increase the copy number. Following purification from *E. coli* cells, plasmids were sequenced with primers (Supplementary Table S4) adjacent to the unique *BamH*I site of YEp13, into which the genomic fragments were cloned. The sequencing results were analysed with the Basic Local Alignment Search Tool (BLAST, NCBI) [29]. The genomic regions contained in the plasmids were identified by comparing with *Saccharomyces* genome database (SGD). As expected, gene fragments containing GRX3 and GRX4 were obtained but were not retained for further analysis. ORFs flanked by upstream and downstream elements were obtained for all positive ‘hit’ gene fragments (Supplementary Table S5) by colony/plasmid PCR and cloned into the *BamH*I site of the high copy-number plasmid pRS425 by Gibson Assembly. Correctness of cloning was confirmed by DNA sequencing and comparison to SGD. The genes responsible for *Δgrx3/4* bypass were functionally confirmed by transforming these plasmid constructs into the shuffle strain, selecting again for leucine prototrophy and counter-selecting on FOA. The ‘hits’ were able to confer growth to the *Δgrx3/4* strain in the absence of the pRS416-Grx3 plasmid.

### Ferric reductase and ferrous iron uptake assays

Standard YPAD plates were modified by addition of 50 µM copper sulfate, in order to repress copper-dependent reductase activity and to permit expression of the iron-dependent reductase. Yeast cells to be tested were spread with cotton swabs forming a patch and grown at 30°C overnight. Top agar was prepared in 10 ml of reductase buffer (50 mM sodium citrate pH 6.5 containing 5% glucose and 0.8% agar). In dark/low light, 100 µM ferric ammonium sulfate and 100 µM bathophenanthroline di sulfonate (BPS) were added to top agar and mixed [30]. The mixture was then poured on top of the YPAD/copper plates, incubated in the dark for 10 min, and then photographed. Ferric reductase activity in the plate assay correlated with the development of a bright red color. Quantitative ferric reductase assays and ferrous iron uptake assays were performed as described earlier [31] with modifications. Briefly, the controls, mutants, and suppressors were grown in appropriate media, defined medium or YPAD containing 50 µM copper sulfate. After growth for 16 h at 30°C, the cultures were diluted into fresh medium of the same composition yielding a density of OD_600_ at 0.2. The cells were allowed to reach exponential growth (3–4 h in rich media or 7–8 h in selective). Then cells were cooled on ice, washed in ice-cold citrate buffer (50 mM sodium citrate, pH 6.5/5% glucose), and assayed. For quantitative ferric reductase assay, 100 μl of the cells were incubated with 1 mM ferric ammonium sulfate and 1 mM BPS for 30 min to 1 h at 30°C in dark/low light, and the reaction was stopped by the addition of 50% trichloroacetic acid. Cells were removed by centrifugation and the *A*_520_ of the ferrous iron–BPS complex was measured. For ferrous iron uptake, 10 μl of cells were added to the 90 μl of iron labeling solution consisting of 1 µM ^55^Fe (50 Ci/g; Amersham Biosciences, Piscataway, NJ, U.S.A.), 1 mM sodium ascorbate and 50 mM sodium citrate. The uptake was allowed to proceed for 1 h at 30°C and was terminated by harvesting the cells on 96-well glass-fibre filters, using a Wallac cell harvester. The cells retained on the filter were washed free of unincorporated iron with water, dried and soaked in a liquid-scintillation fluid (Betalplate Scint; Wallac, PerkinElmer Life Sciences, Boston, MA, U.S.A.). The radioactivity was measured by scintillation counting (Wallac 1450 Microbeta). Results are reported as pmoles/million cells.

### Isolation of yeast mitochondria

Crude mitochondria were isolated from *S. cerevisiae* wild-type strain, mutant and suppressors by the procedure described earlier with modifications [32]. Yeast cells were initially grown in YPAD overnight. Next day, starter culture was inoculated into 1 L media containing of 2% raffinose (YPAR) to induce mitochondrial biogenesis (starting OD_600_ was 0.01 for WT cells/suppressors while 0.1 for *Δgrx3/4* double mutants). When the OD_600_ of cultures reached 2.0–2.5, cells were harvested by centrifugation (5000 ×***g*** for 10 min, RT) and pellet wet weights were measured. Cells were incubated with DTT Buffer (5 ml/g, 0.1 M Tris-SO_4_, pH 9.4 and 10 mM DTT) for 10 min at 30°C. The supernatant was removed by centrifugation (1200 × ***g*** for 10 min, 4°C) and cells were again washed with spheroplast buffer (5 ml/g, 20 mM potassium phosphate, pH 7.4, 1.2 M sorbitol). After this, the cells were incubated with spheroplast buffer (5 ml/g cells) containing 3 mg zymolyase per gram of cells at 30°C for 30 min. At this stage, the spheroplasts were confirmed by microscope, gently centrifuged (2,400 × ***g*** for 10 min, 4°C) to remove the supernatant and washed again with the spheroplast buffer (10 ml/g cells) without disturbing the spheroplasts. From this point forward, experiments were performed on ice. Spheroplasts were resuspended in ice-cold mitochondria buffer (20 mM HEPES-KOH, pH 7.4, 0.6 M sorbitol, 1 mM EDTA, 1 mM phenylmethylsulfonyl fluoride (PMSF) and 1 × protease inhibitor mixture) to a concentration of 2 ml/g cells and then homogenized in a Dounce homogenizer (20 strokes on ice). Homogenate was pooled and centrifuged (1,600 × ***g*** for 10 min at 4°C). The supernatant was saved and centrifuged again as before to remove residual cell debris. Mitochondria were collected from the supernatant by centrifugation (12,000 × ***g*** for 10 min at 4°C to pellet mitochondria). Supernatant was carefully removed, mitochondria pellets were resuspended in 20 mL of mitochondrial buffer and centrifuged (10 min, 12,000 × ***g***, 4°C). The mitochondria pellet was washed again with 1 ml mitochondria buffer using a microfuge tube and finally resuspended in mitochondria buffer omitting EDTA at 2 µl/1 mg mitochondria pellet wet weight. For quantification, 495 µl of 0.6% SDS in Milli Q-water was added into 5 µl of mitochondria, mixed for 15 s on vortex and allowed to sit for 3 min, quickly centrifuged for 10 s, absorbance was measured at 280 nm using a nanodrop (Thermo Scientific).

### Aconitase activity measurement

Aconitase activity was determined using the whole cell extract or isolated mitochondria as described earlier [33]. For the whole cell extract, cells were treated with zymolyase to generate spheroplasts as described above. Spheroplasts or isolated mitochondria were lysed by incubation with 50 mM Tris-HCl, pH 8.0, containing 50 mM NaCl, 1 mM PMSF, 1% Triton X-100, 10% glycerol, 2 mM sodium citrate, and 200 units/ml catalase in a total volume of 50 μl for 30 min on ice. Samples were centrifuged at 15,000 × ***g*** for 10 min at 4°C. Supernatant was mixed with 1/5 volume native gel loading buffer (25 mM Tris-HCl, pH 8.0, 10% glycerol, and 0.025% bromophenol blue) and loaded on a native acrylamide gel composed of a separating gel containing 8% acrylamide, 132 mM Tris base, 132 mM borate, 3.6 mM citrate, and a stacking gel containing 4% acrylamide, 67 mM Tris base, 67 mM borate, 3.6 mM citrate. The running buffer was composed of 25 mM Tris pH 8.3, 192 mM glycine, and 3.6 mM citrate. Electrophoresis was carried out at 180 V at 4°C. The aconitase activity signal was developed by incubating the gel in the dark at 37°C in 100 mM Tris-HCl, pH 8.0, 1 mM NADP^+^, 2.5 mM cis-aconitic acid, 5 mM MgCl_2_, 1.5 mM methylthiazolyldiphenyl-tetrazolium bromide (MTT), 0.3 mM phenazine methosulfate and 5 U/ml isocitrate dehydrogenase [34].

### Microscopy for ribosomal protein localization

The plasmid pRS316-Rpl25-eGFP was designed for the expression of the ribosomal large subunit Rpl25 fused to a GFP reporter. Here, the plasmid was co-transformed with a high copy pRS425 plasmid containing one of the suppressor genes into the Gal-Grx3 *Δgrx4* strain. Then the transformants were grown in defined medium with raffinose but no galactose for 16 h to deplete Grx3. Next, 1 ml of yeast culture was centrifuged (5,000 × ***g*** for 5 min RT) to harvest the cells, washed twice with PBS (5 min each). Cells were fixed in 1 ml of ice-cold 70% ethanol (10 min), washed twice with PBS (5 min each), then stained with 1/3 volume of 4,6-diamidino-2-phenylindole (DAPI) solution (30 μM in PBS buffer for 5 min) and washed with PBS buffer (5 min). Stained cells were diluted by resuspending in 500 µl Milli-Q water. The cells (∼20 µl) were patched on to clean slides and mounted with DAPI-fluromount-G adhesive. The slides were dried overnight (in dark/low light), and images were recorded using a fluorescence microscope (ECLIPSE Ti-E/U/S Nikon) with a 60 × Plan Apo oil DIC H. Recorded images were processed using Image-Pro Plus software. Raw Integrated Density of GFP signal for 100 cells selected randomly with blanking the background was measured using the ImageJ Software.

### Immunoblotting

About 200 µg of mitochondria was resuspended in 20 µl of mitochondrial storage and 5 µl of SDS-PAGE loading buffer. The mixture was sonicated (water bath sonicator for 20 min) and boiled (10 min). Electrophoresis (180 V for 30 min) was performed using SDS-polyacrylamide gel and proteins in gel were transferred to 0.2 μm nitrocellulose membrane. The membranes were blocked with 5% milk powder for at least 1 h followed by the incubation overnight with anti-yeast Cyt-C polyclonal antibody at a concentration of 1:2000 (V/V) (a kind gift from Dr Debkumar Pain). The blots were then incubated in goat anti-rabbit IgG conjugated to horseradish peroxidase (Solarbio Life Science, Beijing, China). Image was developed using a chemiluminescence *in vivo* imaging system (FUSION FX5 VILBER) After imaging, the membrane was stained with 1 × amido black (10 min) to determine the protein traces.

## Results

### Sporulation and tetrad analysis

**Figure 1:**
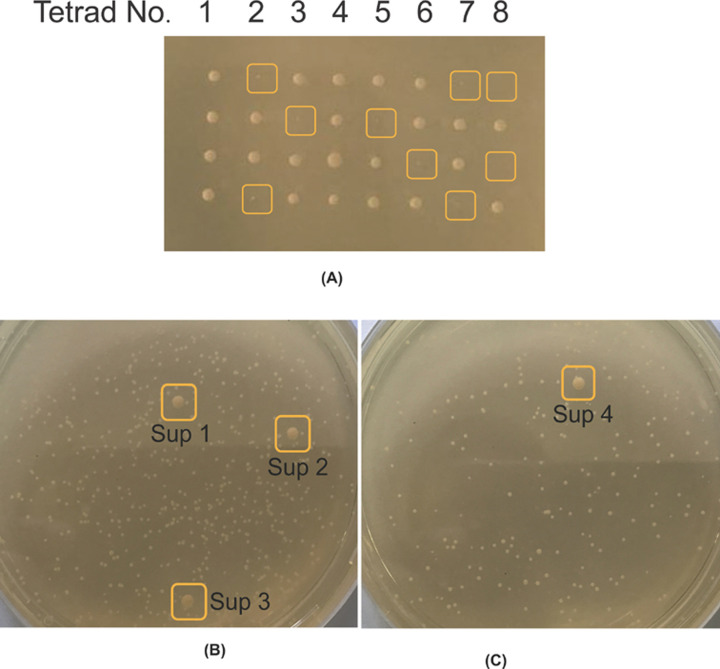
Development of spontaneous suppressors from freshly dissected *Δgrx3/4* tetrad clones (**A**) Tetrad dissection of diploid Grx3/*Δgrx3* Grx4/*Δgrx4.* Each vertical row corresponds to a tetrad from a single ascus (No. 1-8). The tiny clones represent the *Δgrx3 Δgrx4* double mutants (as verified by PCR genotyping) and are highlighted in yellow boxes. Tetrads No. 2, 7 and 8 are NPDs (non-parental ditypes), tetrads No. 3, 5 and 6 are T (tetratypes), and tetrads No. 1 and 4 are PD (parental ditypes). The frequencies are expected from the locations of GRX3 (Chromosome IV) and GRX4 (Chromosome V) on separate chromosomes. (**B** and **C**) Faster growing spontaneous *Δgrx3/4* suppressors appeared among slower growing colonies with a frequency of 1–2%. Boxes labeled with Sup1-4 are four representative suppressors.

Tetrad dissection is a powerful tool in yeast genetics, which allows analysing the products of a single meiosis. It gives information concerning linkage and unique phenotypes associated with double mutants, generally in accordance with the principles of classical Mendelian genetics [25]. Thus, we adopted this method to unambiguously identify the phenotypes associated with *Δgrx3/4* double mutants. A GRX3/*Δgrx3* GRX4/*Δgrx4* diploid strain was grown on nitrogen-limiting medium agar plate to stimulate sporulation. Tetrads were subjected to a carefully timed enzymatic digestion and on a microscopic platform, with the help of a micromanipulator, four haploid spores of each yeast tetrad were selected, separated, and germinated individually on an agar surface (Figure 1A). By comparison with all other genotypes, the double mutant (*Δgrx3Δgrx4*) haploid clones were tiny and came up very slowly. The double mutant genotype was confirmed by PCR and the colonies were transferred to a fresh agar plate (24–48 h at 30°C). A swab of *Δgrx3/4* double mutant cells was then inoculated into rich YPAD liquid medium overnight, cells were harvested by centrifugation and serially diluted, and an average 100–200 cells were uniformly distributed on a YPAD plate in air and observed for the spontaneous appearance of more rapidly growing *Δgrx3/4* suppressors (Figure 1B,C).

### Spontaneous *Δgrx3/4* suppressors restore growth and iron phenotypes of *Δgrx3/4* double mutants

**Figure 2:**
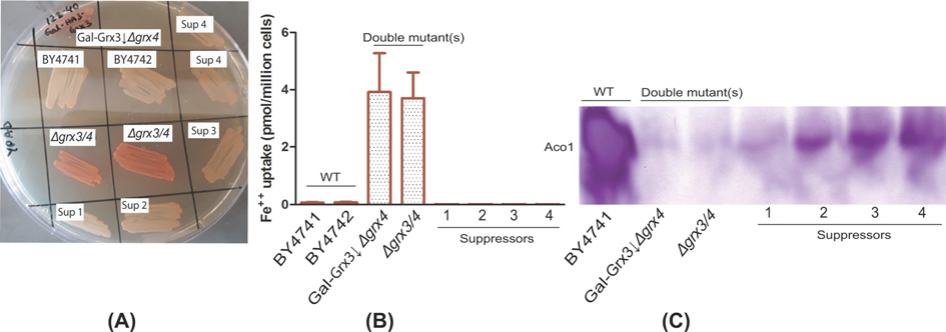
Partial correction of mis-regulated iron metabolism in spontaneous *Δgrx3/4* suppressors (**A**) Plate assay for ferric reductase activities. Red color indicates increased activity, absent in parental strains BY4741 and BY4742, present in *Δgrx3/4* mutants and Gal-Grx3↓ ∆*grx4*, but absent in the spontaneous suppressors Sup 1-4. (**B**) Ferrous iron uptake assays. Markedly increased cellular ferrous iron uptake was seen in the parental Gal-Grx3↓ ∆*grx4* and *Δgrx3/4* strains compared with parental BY4741 and BY4742, but this activity was restored to normal in the suppressors 1-4. For ferrous iron uptake assays, experiments were performed in triplicate and the mean was calculated. Standard deviations are shown by error bars. (**C**) In-gel assays for aconitase activity. Assays were performed on whole cell lysate from BY4741, Gal-Grx3↓*Δgrx4*, *Δgrx3/4* and suppressors 1-4 as described in ‘Materials and methods’. Note high activity in BY4741, absent activity in Grx3/4 depleted or deleted strains, and partially restored activity in the spontaneous suppressors.

*Δgrx3/4* suppressors, identified as more rapidly growing, larger colonies on a background of pinpoint colonies were observed to occur at a frequency of 1–2% when the cultures were grown aerobically (Figure 1B,C). PCR analyses confirmed that the suppressor colonies retained presence of the *Δgrx3/4* deletions. Next, we evaluated iron metabolism. Toward this end, ferric reductase activity, ferrous iron uptake, and aconitase activity were measured. In the spontaneous extragenic suppressors, the iron starvation phenotypes of the *Δgrx3/4* double mutants were alleviated (Figure 2A–C). Ferric reductase (red color in 2A plate assay), repressed in the parental BY4741 and BY4742, induced in the *Δgrx3/4*, was again repressed in the suppressors Sup1, 2, 3, 4 (Figure 2A). Similarly, high affinity cellular ferrous uptake, repressed in the parental BY4741 and BY4742, induced in the Gal-Grx3 *Δgrx4* or *Δgrx3/4*, was again repressed in the suppressors Sup1, 2, 3, 4 (Figure 2B). Aconitase activity, assessed by an in-gel activity assay, present in WT, absent in the Gal-Grx3 *Δgrx4* or *Δ*grx3/4 double mutants, was partially restored in the Sup1, 2, 3, 4 strains (Figure 2C). The suppression effect is relatively modest on aconitase compared with that on cell growth, iron uptake and ferric reductase activity, indicating that minimal Fe-S cluster capacity suffices to maintain normal iron homeostasis and to release cells from growth arrest.

Such suppression could be caused by loss of function mutation in another ORF. Alternatively, the suppressor may act as a genetic dominant, showing penetrance in the *Δgrx3/4* homozygous diploid. Genetic characterization of the suppressors would be of great interest. Thus we had planned to further characterize them by sporulation and tetrad dissection. However, this process was challenging due to extremely slow and uneven growth rate of *Δgrx3/4* double mutants and rapid accumulation of spontaneous suppressor mutation in aerobic growth. This prompted us to look for conditions that avoid suppressor occurrence and/or alternative approaches for suppressor screening.

### Conditions to stabilize growth of *Δgrx3/4* strains: hypoxia or using a shuffling plasmid

**Figure 3:**
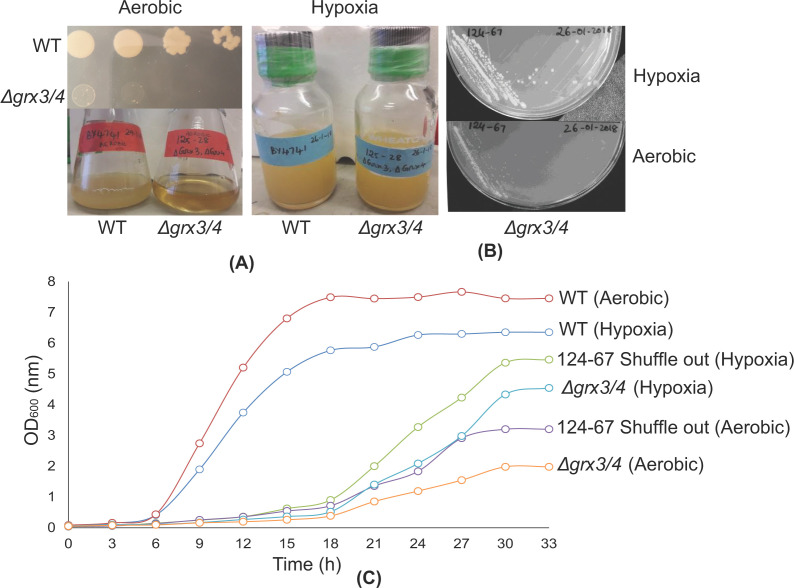
Conditions to improve and stabilize the growth of *Δgrx3/4* (**A**) Growth of *Δgrx3/4* in air is slow, whereas growth under argon is considerably improved. WT and *Δgrx3/4* strains were grown for 16 h in YPAD with aeration and serial 1:5 dilutions of 10^6^ cells were spotted on YPAD agar plates, incubated for 3 days and photographed (upper left). Liquid cultures of WT and *Δgrx3/4* strains were grown with aeration or under argon in YPAD and photographed (left panel in air, right panel under argon). (**B**) Hypoxia allows enhanced growth of the *Δgrx3/4* double mutant. The shuffle strain (*Δ*grx3/4 [GRX3] No. 124-67) was grown on FOA plates for 3 days under aerobic or hypoxic conditions and photographed. (**C**) Growth curves of BY4741 (WT), *Δgrx3/4* obtained from 124-67 lacking the covering plasmid (shuffle out) grown in air (aerobic) or under argon (hypoxic). For comparison a *Δgrx3Δgrx4* strain derived from a freshly dissected tetrad (called *Δgrx3/4*, No. 125-28) was grown aerobically or under hypoxia.

Compared with the WT strain, *Δgrx3/4* mutant exhibited extremely slow and uneven growth rates under aerobic conditions regardless of culture conditions or media composition (Figure 3A). Strikingly, under hypoxic conditions, after bubbling the medium with argon, the growth defect of *Δgrx3/4* mutant was partially rescued, and importantly no suppressor clones were observed indicating improved genetic stability (Figure 3A–C). The fact that hypoxia enhances and stabilizes the growth of the *Δgrx3/4* mutant may have to do with the air-sensitive properties of some critical Fe-S clusters. Perhaps the Aft1/2 and Aft1/BolA clusters, downstream of Grx3/4, might be partially converted to holo forms even in the absence of Grx3/4, and these ‘unauthorized’ Fe/S clusters might be stabilized under hypoxic conditions. Further experiments would be needed to define the mechanism of hypoxic rescue of the *Δgrx3/4* mutant phenotypes.

In a parallel approach we constructed a haploid shuffle strain with the genotype *Δgrx3 Δgrx4*, pRS416-URA3-GRX3-HA3. In this strain, the double mutant phenotype is hidden by the presence of a plasmid-borne copy of GRX3, the latter of which can be removed by counter-selection against the plasmid using fluoroorotic acid (FOA). The shuffle strain following counter-selection grew more slowly than the wild-type in air and was stabilized under hypoxic conditions (Figure 3C), thus resembling the original *Δgrx3/4* strains. The *Δgrx3/4* shuffle strain was useful because it allowed maintenance of healthy growth until unmasking by FOA exposure. The shuffle strain was used for library screen experiments for the identification of *Δgrx3/4* suppressors.

### Genetic screen for *Δgrx3/4* bypass suppressors

**Figure 4:**
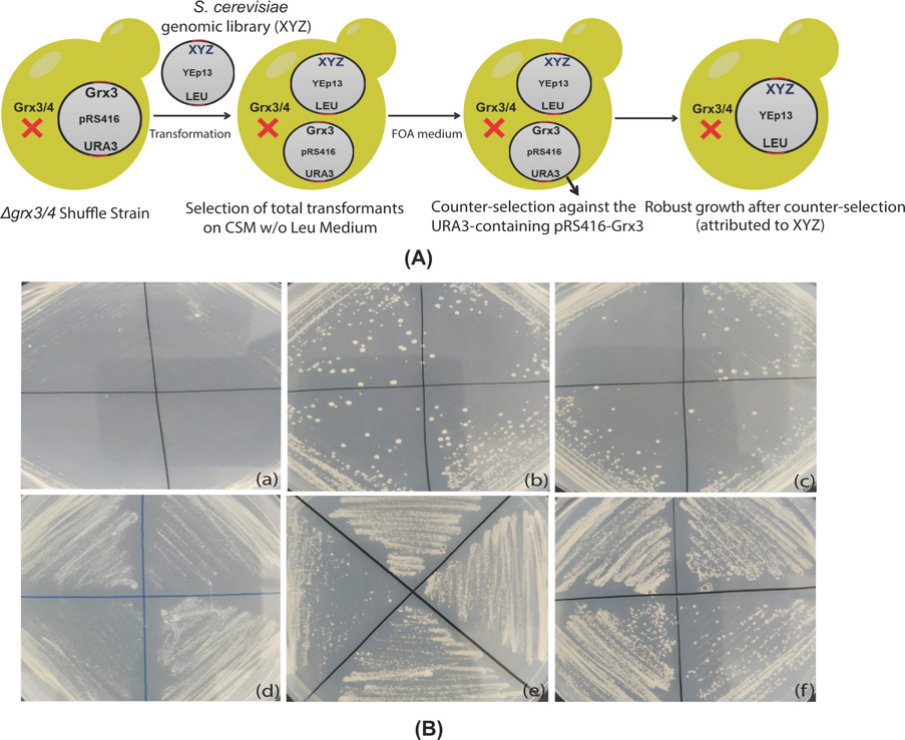
Genetic screen for high copy number suppressors of *Δgrx3/4* (**A**) Schematic illustration of the screening procedure. (**B**) Positive ‘hits’ conferring *Δgrx3/4* bypass in the shuffle strain streaked on each of four quadrants of an FOA plate. Key for the rescuing plasmids: (a) pRS425, (b) pRS425-ESL1, (c) pRS425-ESL2, (d) pRS425-SOK1, (e) pRS425-SFP1, (f) pRS425-BDF2.

**Figure 5:**
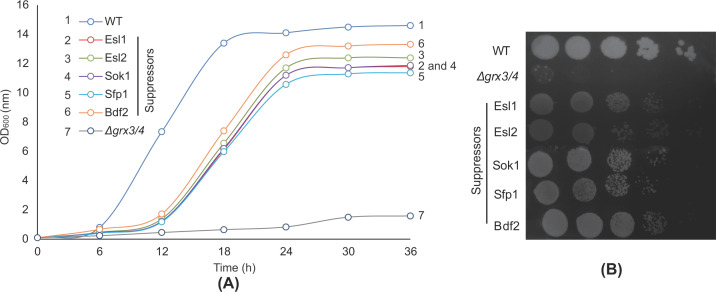
Improved growth of *Δgrx3/4* transformed with high copy-number suppressors (**A**) Growth curves of different strains as labeled (WT, *Δgrx3/4* with pRS425-ESl1, ESL2, SOK1, SFP1, BDF2, pRS425). All cultures were grown in YPAD with initial OD_600_∼0.1 and the growth curves were recorded over 36 h. No selection for the Leu2 containing plasmids was used because the suppressor plasmids self-selected by enhancing growth. (**B**) Serial-dilution colony spotting assays to determine the efficacy of suppressors on restoring *Δgrx3/4* growth.

A YEp13-based yeast high copy-number genomic library (ATCC 37323) was introduced into the yeast shuffle strain (Figure 4A). In a screen of ∼31,000 colonies, 65 positive ‘hits’ were obtained following counter-selection on FOA medium. As expected, many (60 of 65), clones contained either a Grx3 or Grx4 genomic fragment. The plasmids rescued from the remaining 5 clones were sequenced. Each was found to carry a genomic fragment containing several open reading frames (ORFs). To identify the ORFs with suppression activity, each one was cloned along with 500 bp upstream and downstream sequences into pRS425, a 2 μ high copy-number plasmid. Each of five different ORFs (*ESL1, ESL2, SOK1, SFP1* and *BDF2*) alone was found responsible for the suppression activity and able to partially bypass the requirement for Grx3 and Grx4 (Figure 4B). The shuffle strains carrying each suppressor gene (in pRS425) were subjected to FOA counter-selection to remove pRS416-GRX3 before growth analysis. As shown by the growth curves (Figure 5A) and serial-dilution colony spotting assays (Figure 5B), all suppressors were active in partially restoring the growth defects of *Δgrx3/4*.

### Suppressor effects on iron-containing enzymes

**Figure 6:**
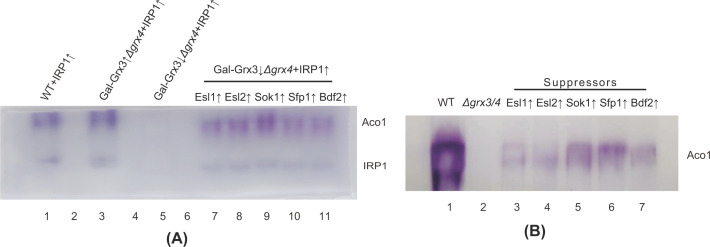
Recovery of cytosolic and mitochondrial Fe-S cluster protein activities in the transformants expressing the suppressors (**A**) In-gel aconitase activity assays with the lysates of 2 × 10^8^ cells separated on an 8% native gel. The Aco1 and IRP1 labeled bands arise from yeast mitochondrial aconitase and heterologous human IRP1 (cytosolic aconitase) expressed from a plasmid, respectively. (**B**) In gel aconitase activity assays with 100 μg mitochondria from BY4742 (WT), *Δgrx3/4* and suppressors separated by an 8% native gel.

Suppressors were then examined for their ability to modify the mutant iron phenotypes. Cytosolic and mitochondrial Fe-S clusters were evaluated by in-gel activity assays for exogenous human cytosolic aconitase (IRP1) and endogenous yeast mitochondrial aconitase (Aco1), respectively [20,34,35]. The two distinct bands on a native gel represent endogenous Aco1 and the heterologous IRP1 (Figure 6A). Grx3, Grx4 deleted (or depleted) cells exhibited complete loss of both cytosolic and mitochondrial aconitase activity, consistent with a previous report of a global defect of the double mutant in Fe-S cluster assembly, affecting both mitochondria and cytoplasmic targets. Partially recovered activities were noted in strains overexpressing Esl1, Esl2, Sok1, Sfp1 and Bdf2 (Figure 6A,B).

**Figure 7:**
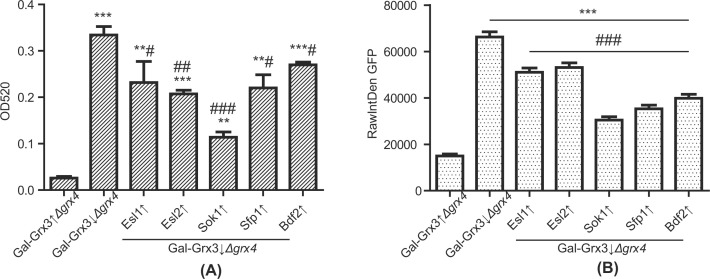

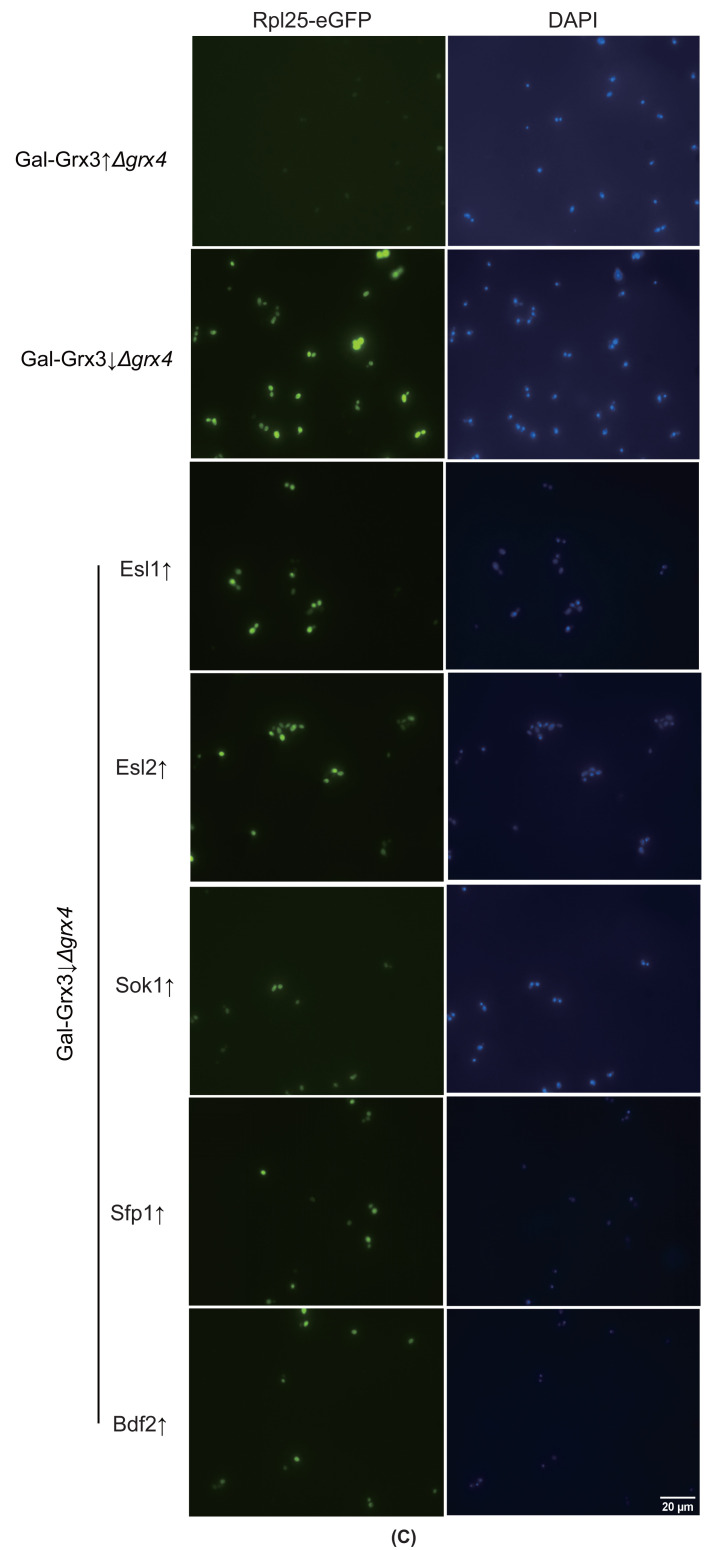
Ferric reductase and nuclear retention of ribosomal protein (Rpl25) (**A**) Ferric reductase quantitative assays. Cells were grown in YPAD, washed with buffered citrate solution at pH6.5 and incubated with 1 mM ferric chloride and 1 mM bathophenathroline disulfonate, after which the OD515 absorbance was assessed. Data are expressed as mean ± SEM (*N* = 6 per group). **P<0.01, ***P<0.001 versus Gal-Grx3↑*Δgrx4*; #*P*<0.05, ##*P*<0.01, ###*P*<0.001 versus Gal-Grx3↓*Δgrx4*. (**B**) Nuclear localization of ribosomal protein Rpl25. Cells were transformed with pRS426-Rpl25-eGFP. Cells were fixed in ice-cold 70% ethanol for 10 min, briefly stained with DAPI and examined using a fluorescence microscope. Raw Integrated Density of GFP signal for 100 cells selected randomly by blanking the background values was measured using the ImageJ Software. Data are expressed as mean ± SEM (*N* = 100 per group). ***P<0.001 versus Gal-Grx3↑*Δgrx4*; ###*P*<0.001 versus Gal-Grx3↓*Δgrx4*. (**C**) Photographs of representative cells to demonstrate nuclear retention of Rpl25. DAPI and Rpl25-eGFP fluorescent images are shown.

Yeast *S. cerevisiae* senses iron starvation by Aft1/2 through Fe-S clusters and then turns on the iron regulon which includes activation of ferric reductase and high affinity ferrous iron transport. Quantitative assays were performed to measure ferric reductase activities of the *Δgrx3/4* cells and the suppressors. Overexpression of each suppressor gene considerably decreased the hyperactivity of ferric reductase in the *Δgrx3/4* strain, with the most pronounced effect observed for Sok1 (Figure 7A). The *Δgrx3/4* mutant was previously reported to retain ribosomal proteins in the nucleus. This defect was attributed to the malfunction of Rli1, an Fe-S cluster protein shuttling between nucleus and cytoplasm, critical for the nuclear export and cytoplasmic assembly of some ribosomal proteins [36,37]. A plasmid carrying Rpl25 with a GFP tag was introduced into the *Δgrx3/4* and corresponding suppressor strains (a gift from Edward Hurt). Consistent with previous observations by others and us [37,38], high fluorescent signals in the nuclei were noticed for Grx3/4-depleted cells. Nuclear fluorescent intensities were statistically much lower for each of the suppressors, indicating partial restoration of function likely through Rli1 partial activation. Among all suppressor genes, Sok1 again appeared to be the strongest effector in correcting Rpl25 subcellular mis-localization (Figure 7B,C).

**Figure 8:**
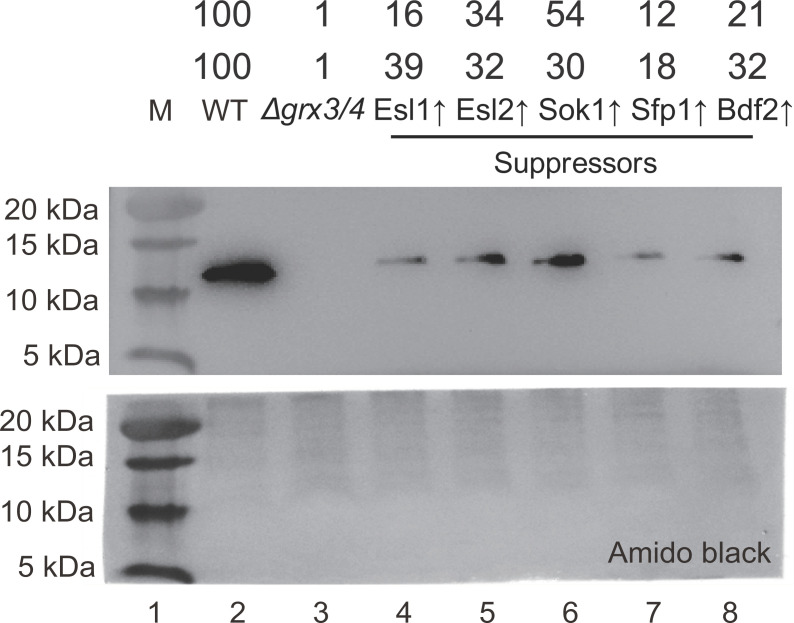
Cyc1 level is partially recovered in the suppressors indicating restoration of heme synthesis Total mitochondrial protein (200 μg) from *Δgrx3/4*, BY4742 (WT), and suppressors were separated by a gradient SDS/PAGE gel (8–16%). After transfer to nitrocellulose membrane, proteins were immunoblotted with the anti-cytochrome c antibody. Amido black staining of the nitrocellulose membrane was performed to demonstrate success of protein transfer and equal loading of each lane. The CytC content relative to the wild-type level for each strain in duplicate experiments is given above the blot.

Consistent with the phenotype of global defects in iron-dependent functions, *Δgrx3/4* cells exhibit heme deficiency [10,39]. The heme cofactor in cytochrome *c* isoform 1 (Cyc1) is necessary to stabilize the apoprotein, and so Cyc1 protein levels in mitochondria provide a surrogate marker for cellular heme levels. Mitochondrial Cyc1 protein levels were analyzed by Western blot. Similar to aconitase activity, partial recovery of Cyc1 level indicating recovered heme synthesis was observed for each of the suppressors, with the highest degree of recovery conferred by Sok1 (Figure 8).

## Discussion

The budding yeast *S. cerevisiae* responds to iron depletion by activation of iron acquisition. At the same time, global reorganization of cellular metabolism occurs, thereby optimizing iron utilization [40]. Such responses require adjustment of the gene expression networks, mediated by transcription, mRNA stability, translation, and protein turnover. Identification of *Δgrx3/4* suppressors (Esl1, Esl2, Sok1, Sfp1 and Bdf2) provides new insights into the global molecular mechanisms involved in the adaptation to iron deprivation.

### ESL proteins and a potential role in pH signaling

Yeast EST/SMG-like (ESL) proteins, Esl1 and Esl2 share extensive sequence and domain topology similarity with human hEST1A/B and Drosophila and *Caenorhabditis elegans* SMG5/6 proteins. Importantly, this similarity comprises the region corresponding to the 14-3-3-like Est-one-homology domain and the C-terminal PIN endonuclease domain with complete conservation of critical D/E residues required for nuclease activity of PIN-domain proteins [41]. In terms of function, Esl1, Esl2 proteins are involved in the expression of a small subset of hexose and amino acid metabolism-related genes via environment-sensing adaptive transcriptional response mechanisms. Reduced genetic fitness of *Δesl1/2* combined with *Δtrf4* (topoisomerase one-requiring function) is also described to deregulate noncoding so-called cryptic unstable transcripts (CUTs) and stable unannotated transcripts (SUTs) in a TRAMP (Trf4/5-Air1/2-Mtr4) complex dependent manner, indicating an anticipated role of these proteins in ribosome biogenesis [42]. Further, the TRAMP complex (a major nuclear exosome cofactor) participates in CTH2 pre-mRNA processing required to post-transcriptionally tune iron-dependent processes as part of the adaptation to iron deprivation [43,44]. In addition, loss of Esl1/2 combined with loss of either of two different components (Dfg16 and Rim8) of the Rim101 pH sensing pathway causes synthetic lethality in yeast. Interestingly, the subset of genes regulated in response to alkaline conditions overlaps considerably with the response to low iron, perhaps due to lowered solubility of iron at high pH [41]. In *Candida albicans*, ferric reductases, FRE2 and FRP1, are regulated by direct interaction with the Rim101p transcriptional factor in alkaline-pH environments [45,46]. In *S. cerevisiae*, Rim101 is proteolytically processed by Dfg16 and Rim8 to facilitate its nuclear translocation and activation of pH-responsive genes under alkaline conditions [47]. Recently RIM9, RIM13 and RIM20 genes, which are involved in the Rim101 pH responsive pathway have been described as genetic interactors of ESL2. Taken together, Esl1/2 may function to bypass defect of iron metabolism either through tuning various intracellular iron utilization processes such as ribosomal biogenesis or through regulating iron availability under alkaline conditions [48]. It is worth noting that the suppression efficiency of Esl1/2 overexpression in the *Δgrx3/4* mutant is the lowest among all newly discovered suppressors in this study suggesting that the suppression mechanism may be less direct.

### Ras-cAMP signaling, SOK1 bypass and restoration of iron regulation

In the yeast *S. cerevisiae*, PKA is the effector protein kinase for the Ras-cAMP pathway, mediating nutrient signals (including iron nutritional signals) and conveying them to cell cycle components. Cells deficient in this activity stop growing and arrest in G1 in a manner resembling that observed for wild-type cells deprived of nutrients [49,50]. PKA has 3 TPK catalytic subunits and a negative BCY1 regulatory subunit. Although they are somewhat redundant with greater than 75% sequence identity, the phosphorylation targets are mostly non-overlapping. Tpk2 was shown to bind to the promoter region of genes encoding ribosomal proteins and in particular regulates genes involved in iron uptake, trehalose degradation and water homeostasis. Further Tpk2, but not Tpk1 or Tpk3, represses transcription of genes involved in high-affinity iron uptake pathway (FRE2, FET3, FTR1 and CCC2). Thus *tpk2* mutants derepress the transport of iron into the cell where it is incorporated into respiratory enzymes that permit growth [51], and in this regard the *tpk2* mutant phenocopies the *grx3/4* mutant. Sok1 (suppressor of kinase) when overexpressed renders a cell independent of PKA function, restoring iron regulation to a *tpk2* mutant. Interestingly Sok1 overexpression also restores iron regulation to a *grx3/4* mutant, suggesting the existence of a parallel pathway for controlling and regulating iron homeostasis. A recent report suggests that Yak1 (PKA-regulated kinase) and Sok1 define a linear pathway that is partially redundant with that of A kinase. Activation of Sok1, by Sok1 overexpression or by inactivation of the negative regulator Yak1, renders a cell independent of PKA function and regulates iron homeostasis [52,53]. It would be interesting to see whether loss of function of Yak1 is active for *Δgrx3/4* suppression.

### The target of rapamycin inhibition bypassed by Spf1 overexpression

The target of rapamycin (TOR) is only found in eukaryotes and is conserved in multiple species from yeast to mammals. The TOR serine/threonine kinase pathway controls cell growth and metabolism in response to environmental signals such as cell integrity, stress, energy status or nutrient levels including iron. The TOR kinase can function in two different complexes, TORC1 and TORC2. Under optimal growth conditions, TORC1 directly phosphorylates the Split Zn-finger transcriptional factor Sfp1, which presumably regulates its nuclear localization for binding to ribosome biogenesis (RiBi) gene promoters to stimulate expression of ribosomal proteins (RPs). However, in response to inhibition of TOR signaling, stress, or changes in nutrient availability, Sfp1 is inactivated by dephosphorylation and exported to the cytoplasm thereby down-regulating the transcription of RP and RiBi genes [54–56]. Studies in different organisms have shown that TOR signaling influences iron metabolism [57]. In yeast, the inhibition of the TOR pathway by Torin2, a potent inhibitor of both TORC1 and TORC2 has recently been shown to increase the expression of metallo reductases involved in iron uptake leading to excess iron sensitivity [58]. Further, upon iron deficiency, yeast cells down-regulate protein synthesis via the TORC1 pathway, repressing the transcription of RP and RiBi genes and the activity of RNA Pol I and III. Our results indicate that Sfp1 overexpression, expected to mitigate low TOR signaling and increased ferric reductase as occurs secondary to iron deficiency, also mitigates effects of *Δgrx3/4* deficiency by restoring reductase regulation and normal growth. The implication is that TOR signaling provides an alternative pathway for the Grx3 Grx4 iron signaling pathway. These results will require further work to clarify the multiple effectors/transcriptional factors that modulate crosstalk between Grx3/4 and TOR signaling.

### Bromodomain factors Bdf1 and Bdf2

*Saccharomyces cerevisiae* contains two bromodomain factors (Bdf1 and Bdf2). They bind to DNA throughout the genome at sites enriched with acetylated histones. Bdf1 appears to be important during early steps of transcription because it recruits the TFIID general transcription factor to acetylated histones 3 and 4 binding to acetyl-lysine in their N-terminal tails. However, cells lacking Bdf1 show an up-regulation of Bdf2 protein levels and redistribution of the acetylated histones at genomic loci normally bound by Bdf1, indicating redundant functions. In general, Bdf1 and Bdf2 are implicated in various aspects of transcription initiation and modulation of chromatin structures, thereby regulating an S-phase stress response [59,60]. Recent data show that various transcription factors are recruited to the chromatin in an iron-responsive manner to modulate DNA replication and repair programs [61]. Thus, Bdf2 overexpression could restore correct iron regulation in presence of *Δgrx3/4* deficiency by reregulating iron-dependent functions. In this regard it would be interesting to ascertain if Aft1/2 DNA loci contain Bfd2 acetylated histones responsive to iron status. Further studies may elucidate how cells coordinate iron metabolism, chromatin structures and DNA replication stress responses.

The suppressors of *Δgrx3/4* do not obviate or bypass the need for iron cofactors as contrasted with some suppressors of *E. coli* suf and isc pathways, that actually live entirely without Fe/S clusters (Takahashi et al., 2015, Mol Micro, 2016 99, 235-848). On the other hand, these high copy suppressors re-established partial Fe/S cluster synthetic activity and iron homeostatic regulation, likely by readjusting various regulatory networks. The various networks we uncovered in our screen apparently shift the regulatory signals from pathways that utilize Fe-S clusters (Grx3/4, Aft1/2) to pathways that do not. The latter include ESL1/2 and pH sensing, ras-cAMP iron-dependent signaling, TOR nutritional responses, bromodomain histone acetylation, each independently able to bypass *Δgrx3/4* and to modulate iron metabolism and cell survival.

## Supplementary Material

Supplementary Figures S1-S2 and Tables S1-S5

## Data Availability

All supporting data in relation to the studies are provided in the manuscript and Supplementary Data. Source data are available from the corresponding authors upon reasonable request.

## Abbreviations

BDF: bromodomain factor
BPS: bathophenanthroline disulphonic acid
CIA: cytosolic sulfur cluster assembly
DAPI: 4,6-diamidino-2-phenylindole
ESL: EST/SMG-like
FOA: 5-fluoroorotic acid
Grx: glutaredoxins
MTT: methylthiazolyldiphenyl-tetrazolium bromide
RiBi: ribosome biogenesis
RPs: ribosomal proteins
SOK: suppressor of kinase
TOR: target of rapamycin
Trx: thioredoxins
