# Supplementary material for: Genetic suppressors of Δgrx3 Δgrx4, lacking redundant multidomain monothiol yeast glutaredoxins, rescue growth and iron homeostasis
Source: Biosci Rep. 2022 Jun 9;42(6):BSR20212665. doi: 10.1042/BSR20212665 (PMC9202360; doi:10.1042/BSR20212665)
Supplement: Supplementary Figures S1-S2 and Tables S1-S5 [file BSR-2021-2665_supp.pdf]

TableS1. List of Strains

Strain number/Name	Genotype	Source
124-67: *Δgrx3/4* shuffle	*MATa Δgrx3::kanMX, Δgrx4::kanMX* [pRS416-URA3-GRX3]	This work
123-40: Gal-Grx3	*MATα his3Δ1, leu2Δ0, lys2Δ0, ura3Δ0, Δgrx4::KanMX* , [His3MX6-PGAL1-3HA-Grx3]	This work
125-28: *Δgrx3/4*	*Δgrx3::KanMX4, Δgrx4::kanMX*	This work
Suppressors 1-4	*MATα Δgrx3/4* suppressors	This work

Table S2. 10 x spore amino acid mix

Amino acid	wt. in mg/g per 500 ml
Adenine	200 mg
Uracil	200 mg
Tyrosine	200 mg
Histidine	100 mg
Leucine	100 mg
Lysine	100 mg
Tyrpophan	100 mg
Methionine	100 mg
Arginine	100 mg
Phenylalanine	500 mg
Threonine	1.75 g
Deionized water	500 mL

TableS3. List of plasmids

Plasmids	Features	Source
YEp13	2μ *S. cerevisiae* AB320 high copy genomic library	ATCC 37323
pRS416-Grx3	This shuffling plasmid is used to cover Grx3 deletion	This work
pRS426-GPDprom-IRP1	2μm *URA3*, GPD promoter driven IRP1	This work
pRS316-RPL25eGFP	*CEN/ARS URA3*	A gift from Eduard Hurt (PMID: 18625724)

Table S4. List of primers

Name	Sequence (5'-3')
Primers used for PCR Analysis of Grx3/4	
Grx3 PF	CGGCCTTCCCTAGCTGAACTAC
Grx3 PR	CATAAATAACATTACCGGCGCGG
Grx4 PF	CCGGAACTTTCCACCAACACCA
Grx4 PR	GCATCACAGGTGCAGCTTGTAC
Kan PR	TCGCAGTGGTGAGTAACCATGC
Primers used for sequencing *S. cerevisiae* AB320 genomic library in YEp13 *E. coli*	
P1	CAGTCCTGCTCGCTTCGCTA

P2	GATATAGGCGCCAGCAACCG
ESL2-PF	GGTGGCGGCCGCTCTAGAACTAGTGAGATGAGGCCTTATTTACTCCC
Name	Sequence (5'-3')
Primers used for cloning genes of interest into pRS425 at *Bam H*1 restriction site by Gibson Assembly	
ESL2-PR	GATATCGAATTCCTGCAGCCCGGGGAAAACTTGCTAGTCTAGCCG
PCK1-PF	GGTGGCGGCCGCTCTAGAACTAGTGAGGCTGCTTAACATTATGGAT
PCK1-PR	GATATCGAATTCCTGCAGCCCGGGGTTGTTGATCCAGTTTCAGTTATTAAAAAAA
RCR2-PF	GGTGGCGGCCGCTCTAGAACTAGTGACTTTGGTGCATGGAGATTAG
RCR2-PR	GATATCGAATTCCTGCAGCCCGGGGCGATGAATTGACTGTTCTGGAC
RAD57-PF	GGTGGCGGCCGCTCTAGAACTAGTGGAACGCTTTCGACTCGGTCC
RAD57-PR	GATATCGAATTCCTGCAGCCCGGGGACAATATTATATTTACTAATTGAACACTTTAGCGA
MAF1-PF	GGTGGCGGCCGCTCTAGAACTAGTGAATCGTGGGCAGTTGCGATA
MAF1-PR	GATATCGAATTCCTGCAGCCCGGGGCATAAAGGTACATAGTTGAAAAGGG
SOK1-PF	GGTGGCGGCCGCTCTAGAACTAGTGTGATTGGTCTCTGCCGTGCG
SOK1-PR	GATATCGAATTCCTGCAGCCCGGGGTACATAATGTGTCTACATTTTATATAGCTG
TRP1-PF	GGTGGCGGCCGCTCTAGAACTAGTGGTAAAATCAACGGTTAACGA

	CAT
TRP1-PR	GATATCGAATTCCTGCAGCCCGGGGGAGATAAGTGTGATAAAGTTTTTACAGC
Name	Sequence (5'-3')
SFP1-PF	GGTGGCGGCCGCTCTAGAACTAGTGGTTCGCTTATAAAGAGAAGGAAAG
SFP1-PR	GATATCGAATTCCTGCAGCCCGGGGATCAGAACAGAAGGAAGTAAGTAAAG
SEI1-PF	GGTGGCGGCCGCTCTAGAACTAGTGAAATTAATTCAATATCAATAATAATATACTATAAGTAACTTAAAAAG
SEI1-PR	GATATCGAATTCCTGCAGCCCGGGGATCATGGGGGAGTAAACTATATCA
AHK1-PF	GGTGGCGGCCGCTCTAGAACTAGTGCGATGGTTTGATCAAAGTACG
AHK1-PR	GATATCGAATTCCTGCAGCCCGGGGATCCTTATGCCCTACCTAAATATAAAC
YET3-PF	GGTGGCGGCCGCTCTAGAACTAGTGATCAAAATGCTCTTGCTCCT
YET3-PR	GATATCGAATTCCTGCAGCCCGGGGTTACTGTCGGCATGAACCTAC
BDF2-PF	GGTGGCGGCCGCTCTAGAACTAGTGAGCACACAGACTTTTAATAATAAAGC
BDF2-PR	GATATCGAATTCCTGCAGCCCGGGGATGTTACAAATCTTTTTTATCCCCAT
CBS1-PF	GGTGGCGGCCGCTCTAGAACTAGTGAACTGACCGAACCTGACCAC

CBS1-PR	GATATCGAATTCCTGCAGCCCGGGGTATAAACAAAAATAAGAAATAAT<br>TGTTTTTACGTACTTAT

Name	Sequence (5'-3')
ESL1-PF	GGTGGCGGCCGCTCTAGAACTAGTGAATCAATCCTTAAAGTGAGAAAGAACA
ESL1-PR	GATATCGAATTCCTGCAGCCCGGGGAATTGTAGAATGGAGTGTTCAAAAAAA
Primers used for sequence analysis of clones constructed on pRS425	
pRS425-P1	CAGGAAACAGCTATGACCATGA
pRS425-P2	TGTAAAACGACGGCCAGT
Primers used for transcriptional analysis of target genes for the wild type and suppressors by qRT-PCR	
BDF2-PF	TTACTGGCAGCACCACAGAG
BDF2-PR	TCGATACTTACCGCGCTGAC
SOK1-PF	TCTTCAACAGGTCCGAGTGC
SOK1-PR	GCAATCGCATTGCCAGTAGG
SFP1-PF	GGGTGGTGTTTCATGGGGAT
SFP1-PF	ACCGTTTGCTGATGCAGGTA
ESL2-PF	GTCCCGAACAGCAACAACAC
ESL2-PR	AGCCTCCATGACGTTTTCGT
ESL1-PF	GCGGGTTCAACAGTGCATTT
ESL1-PR	TCTCCCAATGCAGGCTCAAG

Table S5. The sequencing results of the genomic regions contained in the plasmids isolated from positive hits clones for *Δgrx3/4* bypass.

Screen no	Suppressor colony no	Yeast chromosome no	Genes	Gene(s) responsible for *Δgrx3/4* bypass
01	P1	XI (626728-632950)	ESL2, PCK1	ESL2
01	P4	IV (454206-462595)	RCR2, RAD57, MAF1, SOK1, TRP1	SOK1
02	4A	XII (924141 - 929708)	SFP1, ESI1	SFP1
02	8B	XI (625890 - 632950)	ESL2, PCK1	ESL2
02	13D	IV (325378 - 334774)	BRE1, AHK1, YET3, BDF2, CBS1	BDF2
02	15B	IV (452990 - 462595)	YRB1, RCR2, RAD57, MAF1, SOK1, TRP1	SOK1
02	19D	IX (57093-63174)	ESL1, MCM10	ESL1

Real-time fluorescence quantitative PCR analyses

Total RNAs were extracted from 1-3 mL cultures (~1.2×10^8 cells from each culture) using RNAprep Pure Plant Kit (TIANGEN Beijing) and diluted to 500 ng/μL. 1 μg RNA was reverse transcribed in a 20 μL of reaction mixture containing 0.5 μg random primers and the GoScript Reverse Transcription System according to the manufacturer's recommended protocol (Promega, USA). Obtained cDNA can be frozen at -80 °C for long-term storage. Prior to real-time fluorescence quantitative PCR (qPCR), the reverse transcriptase was inactivated at 70°C for 15 min. In a typical qPCR reaction, 20 μL of reaction mixture contained 4 μL of 5 × diluted cDNA, 0.4 μL of 10 μM gene-specific forward and reverse primers and 10 μL of 2 × Universal SYBR Green Fast qPCR Master Mix, then qPCRs were performed on a Roche LightCycle 96. The primers listed in Table S4 were designed using primer3plus (http://www.primer3plus.com). All reagents and consumables for the experiments were RNase-free.

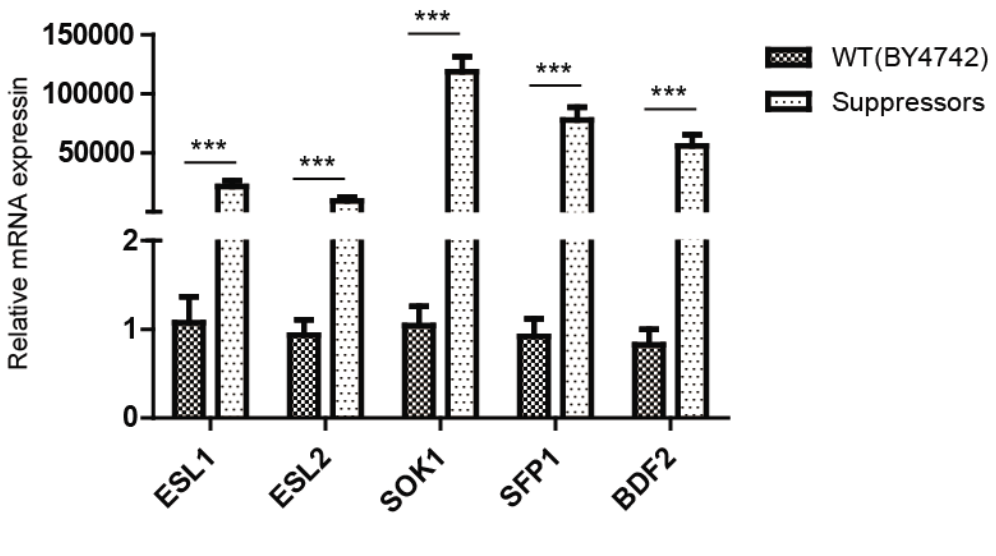

Figure S1. qRT-PCR quantitation of the expression levels of suppressor genes in suppressor strains in relative to the wild type strain. Data are expressed as mean ± SEM (N=3 per group). ***P<0.001 versus WT(BY4742).

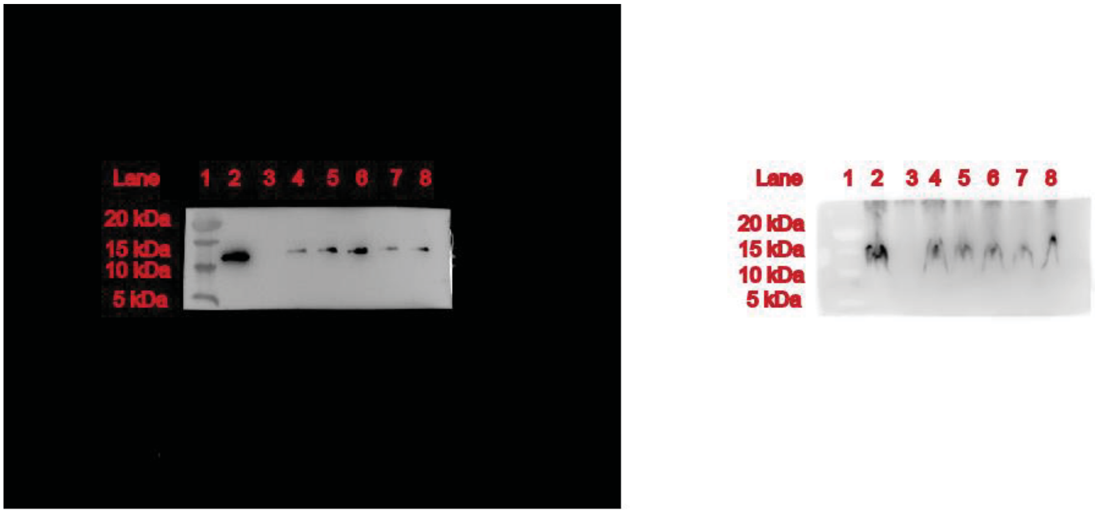

Figure S2. The original images of the CytC western blot. Lane 1-8 correspond to the protein markers, BY4742 (WT), *Δgrx3/4*, and suppressors with overexpression of Esl1, Esl2, Sok1, Sfp1 and Bdf2 respectively. All lanes have been used to build Figure 8.
